# Performance in Olympic triathlon: changes in performance of elite female and male triathletes in the ITU World Triathlon Series from 2009 to 2012

**DOI:** 10.1186/2193-1801-2-685

**Published:** 2013-12-21

**Authors:** Christoph Alexander Rüst, Romuald Lepers, Michael Stiefel, Thomas Rosemann, Beat Knechtle

**Affiliations:** Institute of General Practice and for Health Services Research, University of Zurich, Zurich, Switzerland; INSERM U1093, Faculty of Sport Sciences, University of Burgundy, Dijon, France; Gesundheitszentrum St. Gallen, St. Gallen, Switzerland; Facharzt FMH für Allgemeinmedizin, Gesundheitszentrum St. Gallen, Vadianstrasse 26, 9001 St. Gallen, Switzerland

**Keywords:** Endurance, Performance, Sex difference, Swimming, Cycling, Running

## Abstract

This study investigated the changes in performance and sex difference in performance of the world best triathletes at the ITU (International Triathlon Union) World Triathlon Series (*i.e.* 1.5 km swimming, 40 km cycling and 10 km running) during the 2009-2012 period including the 2012 London Olympic Games. Changes in overall race times, split times and sex difference in performance of the top ten women and men of each race were analyzed using single and multi-level regression analyses. Swimming and running split times remained unchanged whereas cycling split times (*ß* = 0.003, *P* < 0.001) and overall race times (*ß* = 0.003, *P* < 0.001) increased significantly for both women and men. The sex difference in performance remained unchanged for swimming and cycling but decreased for running (*ß* = -0.001, *P* = 0.001) from 14.9 ± 2.7% to 13.2 ± 2.6% and for overall race time (*ß* = -0.001, *P* = 0.006) from 11.9 ± 1.2% to 11.4 ± 1.4%. The sex difference in running (14.3 ± 2.4%) was greater (*P* < 0.001) compared to swimming (9.1 ± 5.1%) and cycling (9.5 ± 2.7%). These findings suggest that (*i*) the world’s best female short-distance triathletes reduced the gap with male athletes in running and total performance at short distance triathlon with drafting during the 2009-2012 period and (*ii*) the sex difference in running was greater compared to swimming and cycling. Further studies should investigate the reasons why the sex difference in performance was greater in running compared to swimming and cycling in elite short-distance triathletes.

## Background

Triathlon is a multi-sports discipline involving swimming, cycling and running and can be held from the Olympic distance (*i.e.* 1.5 km swimming, 40 km cycling and 10 km running) (Bentley et al., 2002) to the Ironman distance (*i.e.* 3.8 km swimming, 180 km cycling and 42.195 km running) such as the ‘Ironman Hawaii’ (Lepers, 2008; Lepers et al., 2013). Recent studies showed that triathlon performances changed over the last decades for both the Olympic distance (Etter et al., 2013) and the Ironman distance (Lepers, 2008; Rüst et al., [Bibr CR22][Bibr CR23]).

Etter et al. (2013) showed for short-distance triathlon at national level during the 2000–2010 period that the overall top five women improved overall race time by ~0.8 min *per annum*, while overall race time remained stable in men. During this period, swimming and running performances remained stable for both women and men while cycling performance decreased significantly by ~0.8 min *per annum* in women and by ~0.5 min *per annum* in men, respectively. Similarly, in a long-distance triathlon at national level such as ‘Ironman Switzerland’ as a qualifier Ironman for the Ironman World Championship ‘Ironman Hawaii’, Rüst et al. (2012a) observed that women improved between 1995 and 2011 in all three split disciplines and overall race times whereas men improved only in the cycling split and in overall race time. In ‘Ironman Hawaii’, the world best elite men improved in the three split times and overall race time, whereas women improved only in cycling, running and overall race time during the last 30 years (Rüst et al., 2012b). An increase in running speed for both ITU (International Triathlon Union) male and female junior elite triathletes has been reported since the introduction of sprint distance events (*i.e.* 750 m swimming, 20 km cycling and 5 km running) at the World Championships 2002 to 2011 (Landers et al., 2013; Vleck et al., 2008). To date, the changes in performance of the world best elite triathletes in short distance triathlon have not been investigated.

The sex difference in triathlon performance has changed during the last decades. For example, the sex difference in overall race time in ‘Ironman Hawaii’ decreased significantly during the last 25 years to stabilize at ~11.3% (Lepers, 2008). During the same period, the sex difference in performance remained quite stable for swimming (~12.5%) and cycling (~12.5%) but it decreased for running from ~13.5% to ~7.3% (Rüst et al., 2012b). At the long-distance duathlon World Championship ‘Powerman Zofingen’ from 2002 to 2011, the sex differences in performance were ~16%, ~17%, ~15%, and ~16% for the 10-km running split, the 150-km cycling split, the 30-km running split and overall race time, respectively (Rüst et al., 2013).

The sex difference in triathlon performance depends upon different variables such as the three disciplines, the distances (*i.e.* short-distance versus long-distance), race tactics (Landers et al., 2008), training (Etxebarria et al., 2013), race experience (Gilinsky et al., 2013), age (Knechtle et al., 2012), anthropometric characteristics (Knechtle et al., [Bibr CR11][Bibr CR12]), and the level of the triathletes (*i.e.* elite versus non-elite) (Lepers et al., 2013). For example, in short-distance triathletes at national level, the sex difference appeared greater for running (~17%) compared to swimming (~15%) and cycling (~13%) (Etter et al., 2013). In contrast for elite long-distance triathletes, the sex difference in performance tended to be lower for running and swimming compared to cycling (Lepers, 2008).

Anthropometric characteristics seem to be important predictors for race time in short distance triathlon at world class level (Landers et al., 2000). Potential reasons in the differences in performance between female and male triathletes are the lower maximum oxygen uptake in women (~52.8 ml · kg^-1^ · min^-1^) compared to men (~61.3 ml · kg^-1^ · min^-1^) (Knechtle et al., 2004), the lower muscle mass in women (~28 kg) compared to men (~41 kg) (Knechtle et al., 2010a), and the higher percent body fat in women (~23.6%) compared to men (~13.7%) (Knechtle et al., 2010a). If we consider the three individual sports, there is a greater difference between the male and female world records for running (*e.g.* 10 km running difference ~ 12.1%, 21.1. km running difference ~12.8%) than swimming (*e.g.* 1,500 m swimming difference ~7.4%) and cycling (*e.g.* cycle hour record difference ~7.9%). The difference between running and the two other disciplines could be explained in part by the biological gender difference in relative body fatness which is higher in women (Landers et al., 1999). Indeed, greater body fat may represent a limit in weight-bearing activities such as running.

In addition, at international level, the differences in performance for elite triathletes between short- and long-distance triathlon might be explained by the possibility of drafting in the cycling split. In international long-distance triathlon, drafting is prohibited in contrast to short-distance triathlons of the ITU World Triathlon Series. Drafting in swimming and cycling may result in a better tactical approach to increase the overall performance in elite Olympic distance triathlons (Bentley et al., 2008). Pacing strategies are observed by elite athletes who are swimming or cycling in a sheltered position inducing several changes of pace (Hausswirth and Brisswalter, 2008). Drafting may alter the sex difference in cycling and in the subsequent running performance. Fast runners seemed to benefit most from drafting during cycling (Hausswirth et al., 1999). For the run split in a short-distance triathlon, an appropriate pacing appeared to play a key role in high-level triathlon performance (Le Meur et al., 2009). Le Meur et al. (2009) showed that both female and male elite triathletes developed specific pacing strategies in running. The men’s running speed decreased significantly over the whole distance whereas women slowed down in the up- and down-hill sections.

Elite short-distance triathletes intending to compete in the Olympic Games need to undergo a qualification in the four years before the Olympic Games. They have to compete in the ITU World Triathlon Series in order to obtain points to qualify for the 55 start places in the Olympic Games. To date, the changes in performance of world class triathletes during a 4-year period before the Olympic Games have not been analyzed. The first aim of the study was therefore to analyze the changes in performance for both elite men and women and the corresponding sex difference in performance in the ITU World Triathlon Series between 2009 and 2012 including the Olympic Games 2012 in London. A second aim was to investigate the sex difference in performance for overall race time and for split times in these athletes.

## Methods

All procedures used in the study met the ethical standards of the Swiss Academy of Medical Sciences and were approved by the Institutional Review Board of Kanton St. Gallen, Switzerland, with a waiver of the requirement for informed consent of the participants given the fact that the study involved the analysis of publicly available data.

### Data sampling and data analysis

The data set for this study was obtained from the website of ITU World Triathlon Series (http://wts.triathlon.org/). Overall race times and split times (*i.e.* 1.5 km swimming, 40 km cycling and 10 km running) over the years in all women and men in the ITU World Triathlon Series between 2009 and 2012 were collected. Races in 2012 were only considered before the Olympic Games. Transition times between swimming and cycling as well as between cycling and running were included in the overall race time. For the first ten women and men in each race, the change in overall race time and split times as well as the sex difference was determined. The sprint distance races (*i.e.* 750 m swimming, 20 km cycling and 5 km running) in the ITU World Triathlon Series were not considered.

### Statistical analysis

In order to increase the reliability of the data analyses, each set of data was tested for normal distribution as well as for homogeneity of variances prior to statistical analyses. Normal distribution was tested using a D’Agostino and Pearson omnibus normality test and homogeneity of variances was tested using a Levene’s test. Regression analysis was used to find significant changes in a variable across years. A hierarchical regression model was used to avoid the impact of a cluster-effect on results in case one athlete finished more than once in the top ten in a race. A Kruskal-Wallis test with Dunn’s test for multiple comparisons was performed after pooling of all data across years to test whether the sex difference differed between the three disciplines. Statistical analyses were performed using IBM SPSS Statistics (Version 19, IBM SPSS, Chicago, IL, USA) and GraphPad Prism (Version 5, GraphPad Software, La Jolla, CA, USA). Significance was accepted at *P* < 0.05 (two-sided for *t*-tests). Data in the text and in the figures are given as mean ± standard deviation (SD).

## Results

Between 2009 and 2012, 58 different women and 55 different men recorded a top ten result in a total of 27 World Triathlon Series event and the 2012 Olympic Games. Eight races were held in 2009, seven races in 2010, eight races in 2011 and four races in 2012 before the 2012 Olympic Games. Table 1 reports the number of finishes during the studied period.

**Table 1:** **Number of top ten finishers and finishes between 2009 and 2012**

Number	Women	Men	Overall
Finishers	58	55	113
Total finishes	270	270	540
1 Finish	13	18	31
2 Finishes	12	9	21
3 Finishes	6	6	12
4 Finishes	6	3	9
5 Finishes	4	0	4
6 Finishes	5	1	6
7 Finishes	2	4	6
8 Finishes	0	1	1
9 Finishes	3	2	5
10 Finishes	1	1	2
>10 Finishes	6	10	16

**Figure 1:**
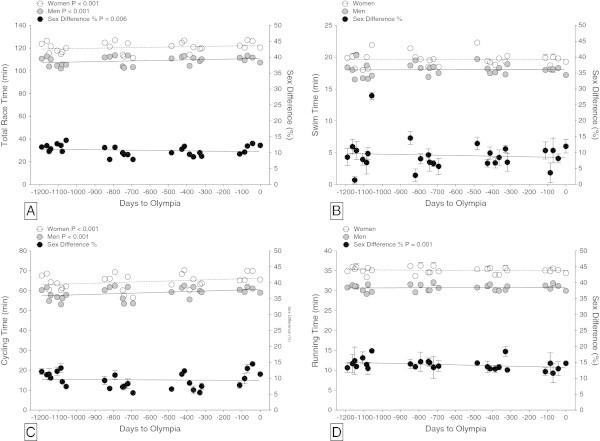
**Change in performance at the ITU World Triathlon Series with corresponding sex differences from 2009 to 2012.** Overall race time **(Panel A)**, swimming **(Panel B)**, cycling **(Panel C)** and running time **(Panel D)**. The time line is expressed in days before the Olympic Games.

**Figure 2:**
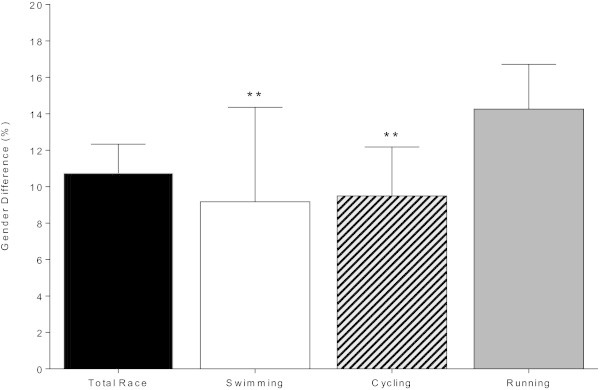
**Mean (±SD) sex difference in swimming, cycling, running and overall race times for the 2009–2012 period.** ** = *P* < 0.01; significantly different from running.

Figure 1 shows the changes in overall race times and split times across all 28 races. For women and men, the split times in swimming and running remained unchanged. The cycling split times increased for women from 63.5 ± 3.0 min to 67.2 ± 2.3 min and for men from 57.4 ± 2.6 min to 60.4 ± 1.1 min (Table 2) also when corrected for multiple finishes (Table 3). Overall race times increased for women from 119.5 ± 3.4 min to 123.3 ± 2.9 min and for men from 106.9 ± 3.6 min to 110.6 ± 2.2 min (Table 2) also when corrected for multiple finishes (Table 3). The sex difference in performance remained unchanged for swimming and cycling but decreased for running from 14.9 ± 2.7% to 13.2 ± 2.6% and for overall race time from 11.9 ± 1.2% to 11.4 ± 1.4% (Table 2) also when corrected for multiple finishes (Table 4). The sex difference in running (14.3 ± 2.4%) was significantly (*P* < 0.001) greater compared to the sex difference in swimming (9.1 ± 5.1%) and cycling (9.5 ± 2.7%) (Figure 2).

**Table 2:** **Mean values ± SD of swimming, cycling, running and total time for both women and men at the ITU World Triathlon Series with corresponding sex difference for each discipline**

Women	2009	2010	2011	2012
Total time (min)	119.5 ± 3.4	119.3 ± 6.0	121.1 ± 3.8	123.3 ± 2.9
Swimming split time (min)	19.6 ± 1.3	19.6 ± 1.0	19.9 ± 1.1	19.5 ± 0.6
Cycling split time (min)	63.5 ± 3.0	63.2 ± 4.7	65.0 ± 3.2	67.2 ± 2.3
Running split time (min)	35.2 ± 0.9	35.2 ± 1.1	35.0 ± 0.9	35.1 ± 0.7
**Men**				
Total time (min)	106.9 ± 3.6	108.7 ± 4.8	1101. ± 2.7	110.6 ± 2.2
Swimming split time (min)	17.8 ± 1.3	18.2 ± 0.8	18.2 ± 0.8	17.9 ± 0.5
Cycling split time (min)	57.4 ± 2.6	58.5 ± 3.8	60.0 ± 2.2	60.4 ± 1.1
Running split time (min)	30.6 ± 0.9	30.8 ± 1.0	30.7 ± 0.7	31.0 ± 0.9
**Sex difference**				
Total time (%)	11.9 ± 1.2	9.7 ± 1.5	10.0 ± 1.1	11.4 ± 1.4
Swimming split time (%)	10.6 ± 7.5	7.8 ± 3.9	9.0 ± 2.9	9.1 ± 4.0
Cycling split time (%)	10.8 ± 2.1	7.9 ± 2.0	8.3 ± 2.5	11.3 ± 2.6
Running split time (%)	14.9 ± 2.7	14.4 ± 2.1	14.1 ± 2.2	13.2 ± 2.6

**Table 3:** **Multi-level regression analyses for change in performance across years for women and men (Model 1) with correction for multiple participations (Model 2)**

Model	***ß***	SE ( ***ß*** )	Stand. ***ß***	T	***p***
Swim split women					
1	−3.442 e^-005^	0.000	−0.013	−0.209	0.835
2	−3.442 e^--005^	0.000	−0.013	−0.209	0.835
Swim split men					
1	8.277 e^--005^	0.000	0.034	0.562	0.575
2	8.277 e^--005^	0.000	0.034	0.562	0.575
Bike split women					
1	0.003	0.001	0.292	5.003	<0.001
2	0.003	0.001	0.292	5.003	<0.001
Bike split men					
1	0.003	0.000	0.358	6.277	<0.001
2	0.003	0.000	0.358	6.277	<0.001
Run split women					
1	0.000	0.000	−0.081	−1.338	0.182
2	0.000	0.000	−0.081	−1.338	0.182
Run split men					
1	0.000	0.000	0.082	1.348	0.179
2	0.000	0.000	0.082	1.348	0.179
Overall race time women					
1	0.003	0.001	0.227	3.820	<0.001
2	0.003	0.001	0.227	3.820	<0.001
Overall race time men					
1	0.003	0.001	0.312	5.383	<0.001
2	0.003	0.001	0.312	5.383	<0.001

**Table 4:** **Multi-level regression analyses for change in sex difference across years for women and men (Model 1) with correction for multiple participations (Model 2)**

Model	***ß***	SE ( ***ß*** )	Stand. ***ß***	T	***p***
Swim split					
1	−0.001	0.001	−0.081	−1.324	0.187
2	−0.001	0.001	−0.081	−1.324	0.187
Bike split					
1	0.000	0.000	−0.044	-.716	0.474
2	0.000	0.000	−0.044	-.716	0.474
Run split					
1	−0.001	0.000	−0.210	−3.511	0.001
2	−0.001	0.000	−0.210	−3.511	0.001
Overall race time					
1	−0.001	0.000	−0.167	−2.772	0.006
2	−0.001	0.000	−0.167	−2.772	0.006

## Discussion

The aim of this study was (*i*) to analyze the changes in performance and sex difference for both elite women and men in the ITU World Triathlon Series between 2009 and 2012 including the Olympic Games 2012 in London and (*ii*) to investigate the sex difference in performance for overall race time and split times in these athletes. The present findings showed an increase in overall race times and cycling split times between 2009 and 2012 for the top ten finishers in the ITU World Triathlon Series including the 2012 Olympic Games.

However, due to several limitations, *e.g.* different races each year, different environmental conditions, and potential differences in the course lengths, these changes in cycling and total performance across the period 2009–2012 may be not relevant. Additionally, in most cases, the female and male events were not only held at different times of the day but also on different days. Besides these limitations, the most interesting findings were (*i*) a decrease in the sex difference for running and overall race time with a stabilization in swimming and cycling and (*ii*) a greater sex difference in running compared to cycling and swimming performances.

### Decrease in sex difference in running and total time performance

Interestingly, the results showed that the sex difference in performance decreased for running and overall race time but remained unchanged for swimming and cycling. By comparison, at the Ironman World Championship ‘Ironman Hawaii’ between 1983 and 2012, the sex difference for the annual top ten remained unchanged for swimming and cycling at ~12.5% but decreased in running from ~13.5% to ~7.3% and in overall race time from ~15.2% to ~11.3% (Rüst et al., 2012b). For short-distance triathletes competing at national level between 2000 and 2010, the sex difference for the annual top five remained unchanged for swimming (~15.2%), cycling (~13.4%), running (~17.1%), and overall race time (~14.8%) (Etter et al., 2013). The present short-distance triathletes competing at international level reduced, however, the sex difference in running and overall race time between 2009 and 2012. The decrease in sex difference in overall race time might be due to the decrease in sex difference in running. However, the decrease in sex difference in running over the 2009–2012 period is difficult to explain and may be linked to different approaches of training in women and different race strategies (*e.g.* more energy saved in the cycling split).

The present results showed that the values of sex difference in Olympic distance triathlon performance for international elite triathletes were lower compared to those found for elite triathletes competing at national level (Etter et al., 2013). The sex differences were 9.1 ± 5.1% *versus* 15.2 ± 4.6% for swimming, 9.5 ± 2.7% *versus* 13.4 ± 2.3% for cycling, 14.3 ± 2.4% *versus* 17.1 ± 2.5% for running and 10.7 ± 1.6% *versus* 14.8 ± 1.8% for overall race time. The sex difference in cycling time was significantly (*P* < 0.001) lower than for swimming and running (Etter et al., 2013).

These findings suggest that international female short distance triathletes tended to reduce the gap with men. During the studied period, the sex difference varied between the disciplines with greater sex differences in running compared to cycling and swimming. In contrast, there was a smaller sex difference in running compared to cycling in elite long-distance triathletes in ‘Ironman Hawaii’ (Rüst et al., 2012b). This discrepancy in sex difference in running between short and long-distance triathlon is intriguing. However, the finding could be linked to the fact that in international long-distance triathlons such as Ironman triathlons drafting is forbidden in contrast to short distance triathlon where drafting is allowed. Pacing and drafting is essential for the race outcome in a short distance triathlon (Landers et al., 2008; Vleck et al., 2008). For example, the winner in a draft legal Olympic distance triathlon exited the water in the first pack in 90% of elite male and in 70% of elite female racers (Landers et al., 2008).

### Greater sex difference in running compared to cycling and swimming

The results showed that in the 28 considered races of the ITU World Triathlon Series 2009–2012 including the 2012 Olympic Games, the sex difference was higher in running (~14.3%) compared to swimming (~9.1%) and cycling (~9.5%). Similar findings have been reported for recreational short-distance triathletes competing at national level (Etter et al., 2013) and non-elite Ironman triathletes (Lepers and Maffiuletti, 2011) where the sex difference in performance was greater in running compared to swimming and cycling.

However, in the present athletes at world class level, the absolute value of the sex difference in swimming was lower (~9.1%) compared to recreational short-distance triathletes with 15.2 ± 4.6% and elite Ironman triathletes with 12.1 ± 1.9%. Also for cycling and running, the absolute values of the sex difference were lower in the ITU World Triathlon Series (9.5 ± 2.7% and 14.3 ± 2.4%, respectively) compared to recreational short-distance triathletes at national level (14.3 ± 2.4% and 17.1 ± 2.5%, respectively) (Etter et al., 2013) and elite Ironman triathletes (15.4 ± 0.7% and 18.2 ± 1.3%, respectively) (Rüst et al., 2012b). It seemed that the sex difference in performance in international level triathlons tended to be lower compared to triathlons at national level (Etter et al., 2013).

The higher sex difference in running compared to swimming and cycling might be due to drafting during the swimming and cycling splits. It has been shown that drafting can improve the performance in the subsequent split discipline in a short distance triathlon. Drafting during the swimming (Delextrat et al., 2003) and cycling part (Hausswirth et al., 1999,2001) might select for a fast running split time in a short-distance triathlon. Hausswirth et al. (2001) showed that drafting continuously behind a lead cyclist allowed triathletes saving a significant amount of energy during the bike leg of a sprint triathlon and created the conditions for an improved running performance. Fast runners seemed to benefit most from drafting during cycling (Hausswirth et al., 1999). For the running split in short-distance triathlon, an appropriate pacing appeared to play a key role in high-level triathlon performance (Le Meur et al., 2009).

Differences in drafting between the sexes might be essential for race outcome. Male triathletes might benefit more cycling drafting then women because they tend to ride in larger packets (Landers et al., 2008). The men’s running speed decreased significantly over the whole distance whereas women slowed down in the up- and down-hill sections (Le Meur et al., 2009). Vleck et al. (2006) demonstrated that both women and men in an elite Olympic distance triathlon ran faster over the first 993 m than most other run sections. However, no clear benefit of this strategy was apparent. In addition to drafting, the tactical approach in triathletes might be different. Vleck et al. (2006) showed that an inferior swimming performance may result in a tactic involving greater work in the initial stages of the cycling split which may substantially influence subsequent running performance. Because sex differences in running performance are greater for running compared to swimming and cycling, it could be suggested that elite male triathletes benefit more from cycling drafting than female athletes.

## Conclusion

During the 2009–2012 period, the world’s best female short-distance triathletes reduced the gap with male athletes in running and total triathlon performance. The sex difference in performance was greater for running (~14%) compared to swimming (~9%) and cycling (~10%). The influence of cycling drafting on running performance may differ between elite female and male triathletes. Future studies are required to clarify why the sex difference in running is greater compared to swimming and cycling in international short distance triathlon races with drafting.
